# Descemetic and Predescemetic DALK in Keratoconus Patients: A Clinical and Confocal Perspective Study

**DOI:** 10.1155/2014/123156

**Published:** 2014-08-26

**Authors:** Domenico Schiano-Lomoriello, Rossella Annamaria Colabelli-Gisoldi, Mario Nubile, Francesco Oddone, Giorgio Ducoli, Carlo Maria Villani, Leonardo Mastropasqua, Augusto Pocobelli

**Affiliations:** ^1^IRCCS Bietti Foundation, Via Livenza 3, 00198 Rome, Italy; ^2^Azienda Ospedaliera San Giovanni-Addolorata, Rome, Italy; ^3^Universita degli Studi “Gabriele D'Annunzio”, Chieti, Italy

## Abstract

*Purpose*. To evaluate the clinical outcomes and in vivo confocal microscopy (IVCM) features of keratoconus patients who underwent deep anterior lamellar keratoplasty (DALK). *Methods*. DALK was performed using the big bubble technique in all the patients. If the bubble was not successful to bare the descemet membrane, a manual dissection layer-by layer was performed to expose a deep stromal plane close to the DM. The patients were divided in two groups depending on the intraoperative baring of the descemet membrane: predescemetic DALK (PD-DALK) and descemetic DALK (D-DALK) group. *Results*. One month after surgery the D-DALK patients show an increase of mean BCVA. In the PD-DALK group mean BCVA did not show significant improvement as compared to preoperative values. At 6 months after surgery mean BCVA was found to be similar in both groups. At 1 month IVCM the peak of reflectivity of the interface was lower in D-DALK group compared to PD-DALK. At 6 months the values of reflectivity were comparable. *Conclusions*. At 1 month D-DALK seems to lead to a minor interface reflectivity and to a better BCVA; these differences disappear after 6 months and the values of interface reflectivity and BCVA are comparable between D-DALK and PD-DALK.

## 1. Introduction 

Deep anterior lamellar keratoplasty (DALK) is a lamellar corneal graft technique, alternative to penetrating keratoplasty (PK), in patients with healthy endothelium. In this technique, the donor cornea is transplanted free from endothelium and descemet's membrane (DM) [1, 2]. The recipient bed after stromal dissection, aimed at removing the diseased stroma, generally consists of endothelium and DM and sometimes a thin layer of rear stroma [1].

The advantages of DALK compared to PK include a reduced risk of endophthalmitis, expulsive haemorrhage and anterior chamber lesions, with DALK being an extraocular surgical procedure [3]. Furthermore the preservation of the host endothelium reduces the risk of corneal endothelial failure due to endothelial cell loss or rejection [4].

Corneal transparency is a key factor influencing the visual outcome both in PK and DALK, but in the latter visual recovery is also significantly affected by the light scattering occurring at the donor host interface and by the amount and regularity of residual stroma adherent to DM [5, 6]. Different surgical techniques have been proposed to improve the postoperative donor-host interface quality, with the aim of minimizing recovery time while maximizing visual recovery.

In the predescemetic DALK technique (PD-DALK), a thin variable portion of residual stroma is left on the recipient bed after stromal dissection, while in the descemetic DALK (D-DALK) there is a complete stromal excision with total exposure of DM on which the donor button is directly placed. One of the most popular surgical methods to obtain a D-DALK is the big bubble technique [5] which allows the cleavage separation between DM and posterior stroma by forceful injection of air into the deep stroma. The overall success in separating the DM from the rear stromal tissue by using this technique has been reported to be approximately 70–80% worldwide [3].

When the big bubble is not obtained, an intrastromal manual dissection has to be performed excising the stroma layer by layer, until a regular deep stromal plane, close to the endothelium, is created in order to obtain a PD-DALK [7, 8].

A recent report showed that D-DALK and PD-DALK have comparable visual acuity and refractive outcomes, biomechanical properties, corneal thicknesses, and endothelial cell densities [8].

Moreover, it has been also reported that D-DALK, performed for variable indicating pathologies, is associated with less keratocytes activation, at confocal microscopy evaluation, and less interface haze, as observed subjectively at the slit lamp examination, despite the fact that no differences in mid-term visual outcomes were detected in comparison with PD-DALK [8].

The purpose of this study was to evaluate the morphological characteristics of donor-host interface, by using confocal microscopy, and the corresponding visual outcome after PD-DALK and D-DALK for keratoconus.

## 2. Methods

Consecutive patients suffering from keratoconus with healthy endothelium requiring keratoplasty were enrolled in this prospective comparative observational study.

The study followed the tenets of the Declaration of Helsinki and had local ethical committee approval. An informed consent was obtained from all participants.

Surgeries were performed in general anesthesia using the same surgical technique (DALK with big bubble described by Anwar and Teichmann) in all patients [5].

If a complete baring of the descemet membrane was achieved, the procedure was classified as successful D-DALK; while in case of failure in exposing the DM, a predescemetic stromal delamination was performed manually and the procedure was classified as PD-DALK.

Visual acuity, corneal topography, confocal microscopy, and endothelial cell count were evaluated in all the patients.

Preoperative ocular examinations included evaluation of Snellen best corrected visual acuity (BCVA), complete ophthalmological examination with biomicroscopy of the anterior segment, and fundus examination. Preoperative endothelial cell count was performed in each eye using specular microscopy (Cell Chek XL-Konan, Irvine, CA, USA). The mean of 3 consecutive endothelial cell density measurements, obtained performing a manual cell count within a standardized region of interest (0.1 mm^2^) in the central cornea, was used for analysis.

Postoperative ocular examinations included BCVA, slit lamp biomicroscopic examination, endothelial cell density analysis using specular microscopy, corneal topography (Magellan Mapper-Nidek Technologies, Padova, Italy), and in vivo confocal microscopy (IVCM) (Confoscan 4-Nidek Technologies, Padova, Italy) to characterize the interface and stromal morphology.

Postoperative examinations were performed at 1 and 6 months after surgery.

### 2.1. Surgical Technique

**Figure 1:**
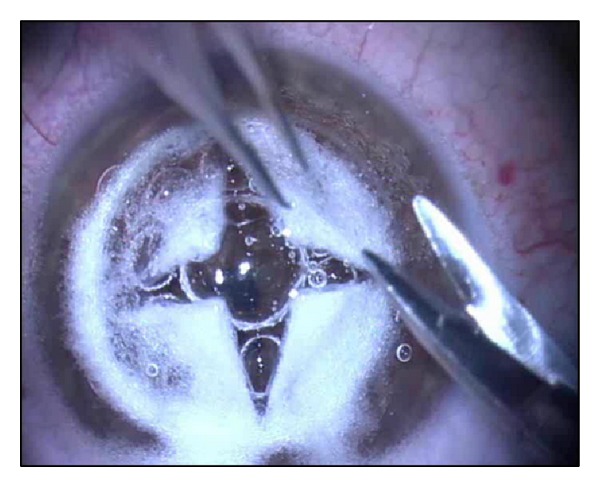
D-DALK: the Descemet's membrane is exposed after the air-bubble creation and blunt-tipped Vanna's scissors were used to excise the deepest stromal layers, cut into four parts.

All patients were operated on by one surgeon with a long standing experience in corneal surgery (AP). DALK was performed under general anaesthesia using the big bubble technique previously described by Anwar and Teichmann [5]. A partial thickness trephination of 8.0 mm in diameter, aiming to include the entire cone, was made using the Hanna trephine (MORIA, Paris, France). The trephine was regulated to obtain a cut of 400 microns in depth into the corneal stroma. Using a Fogla (Bausch and Lomb-Storz, Rochester, NY, USA) pointed dissector a pocket aimed at facilitating the entrance of the tip of the cannula into the stroma was created and then a Fogla 27 Ga (Bausch and Lomb-Storz Rochester, NY, USA) air injection cannula to a 5 mL syringe was used to inject the air into the deep stroma to obtain the “big bubble” air separation between the stroma and the descemet's membrane. A paracentesis of the anterior chamber was made to control the intraocular pressure. Air was introduced into the anterior chamber to verify the presence of a big bubble. When the big bubble was obtained, a partial-thickness anterior keratectomy was performed using a crescent knife (ALCON Laboratories, Texas, USA). The residual stromal cap delimitating the air bubble was opened using a 15° slit-knife (ALCON Laboratories, Texas, USA) to create a linear incision in the center of the cornea. Using a blunt-tipped Vanna's scissors, the deepest stromal layers were cut into four parts that were excised to expose the descemet's membrane (D-DALK) (Figure 1).

Conversely, if the descemet membrane was not reached, a manual dissection layer by layer with a blunt-tipped spatula (Janach, Como, Italy) was performed to expose a regular deep stromal plane close to the DM (PD-DALK).

In all the cases a 31°C organ culture donor cornea of identical size (8.0 mm) was trephined; the endothelium was stained by Trypan Blue (ALCHIMIA srl, Padova, Italy) and removed with methylcellulose sponges (ALCON Laboratories, Texas, USA). The donor cornea was sutured onto the recipient bed with a single running 10-0 nylon suture (ALCON Laboratories, Texas, USA). The anterior chamber was then filled with a balanced saline solution (BSS) and the air was removed. At the end of the surgery netilmicin 0.4% and dexamethasone 0.1% eye drops were given.

After operation, all patients received a topical fixed combination of netilmicin 0.4% and dexamethasone 0.1% 6 times a day.

These eye drops were tapered within the following 3 months after surgery.

Suture adjustment was performed when topographic astigmatism was superior to 3.5 diopters within the third and fourth postoperative weeks. In case of regression of the effect, postoperative adjustment was repeated for a maximum of three times until 3 months after operation. Suture removal was performed between 10 and 12 months after operation.

### 2.2. In Vivo Confocal Microscopy

All patients underwent IVCM examination at 1 and 6 months after surgery by using a white light—scanning slit confocal microscope (Confoscan 4-Nidek Technologies, Padova, Italy). This instrument is equipped with an Achroplan (Zeiss, Oberkochen, Germany) nonapplanating ×40 immersion objective lens designed for full-thickness examination of the cornea, with a working distance of 1.92 mm and a motorized focusing device. The center of the cornea was studied during all examinations.

Each examination included two complete confocal acquisitions of the entire central cornea for each eye with a total examination time of less than 5 minutes. The main parameters for the sequence acquisition were set using 3 passes, for each complete corneal examination and a *z*-axis movement range of 1000 *μ*m, giving a theoretical *z*-axis distance between images in the scans of 10 *μ*m. The position on the *z*-axis of the corneal thickness of each image was obtained using the Z-scan function of the instrument. The Z-scan is a graph showing the depth coordinate (expressed in micrometers) on the *z*-axis and the level of reflectivity (expressed in arbitrary numerical units, called light reflectance units (LRU)) on the *y*-axis for each corneal image included in the scan [9, 10].

In all the cases IVCM allowed us to observe the endothelial cells, the integrity of the descemet membrane, and the interface morphological characteristics.

The donor-host stromal interface was defined as the corneal sublayer located in the posterior stroma (or adjacently to the DM) with evident discontinuity of the stromal keratocyte and extracellular matrix architecture and nonhomogeneous reflectivity.

The donor-host stromal interface reflectivity was calculated by IVCM as the average of 3 peaks of values obtained by 3 reliable Z-scan graphics and expressed in light reflectivity units (LRU) provided by the instrument's software.

Residual bed thickness was calculated as the distance between the endothelial layer peak and the interface reflectivity peak. Each value of residual bed thickness was considered as the average of 3 measurements obtained from 3 reliable Z-scan graphics.

### 2.3. Statistical Analysis

Continuous data have been described as mean ± standard deviation and categorical or nominal data as frequencies. Between groups, differences in the behaviour of the variables of interest over time have been analyzed by MANOVA for repeated measures. Time point differences were tested by paired* t*-test or Wilcoxon's sign rank test as appropriate for within-group comparisons and by independent *t*-test or Mann-Whitney *U* test as appropriate for between-groups comparisons. Linear regression analysis was used to explore relationships between variables. *P* values < 5% were considered as statistically significant.

## 3. Results

Thirty eyes of 30 patients were included in the study, and in 18 eyes (60%) a successful D-DALK with complete baring of the DM was obtained, while in 12 cases (40%) a thin stromal layer was left overlying the host DM (PD-DALK). The two groups of patients did not show demographic and preoperative clinical differences. No cases of intraoperative rupturing of DM or conversion into PK occurred and no stromal rejections were detected throughout the study. No other intraoperative or postoperative complications were recorded.

Endothelial cell count, evaluated by specular microscopy, was 2265 ± 429 and 2136 ± 388 cell/mm^2^ before surgery in the D-DALK and PD-DALK groups, respectively (*P* = 0.4). Cell count did not show significant changes over the follow-up time in either groups (*P* = 0.95). One month after surgery endothelial cell count was 2227 ± 380 in the D-DALK group and 2118 ± 36 cell/mm^2^ in the PD-DALK group (*P* = 0.27) and 6 months after surgery it was 2254 ± 328 in the D-DALK group and 2134 ± 373 cell/mm^2^ in the PD-DALK group (*P* = 0.37), respectively.

Baseline mean BCVA was 0.32 ± 0.18 in the D-DALK group and 0.29 ± 0.17 in the PD-DALK group (*P* = 0.6). Visual acuity recovery time was significantly different between the two groups. At one month after surgery the mean BCVA significantly improved up to 0.55 ± 0.18 (*P* = 0.0004), with respect to preoperative values in the D-DALK group. Differently in the PD-DALK group at one month after surgery BCVA did not show significant improvement as compared to preoperative values (0.31 ± 0.07; *P* = 0.69). At 6 months after surgery mean BCVA was found to be similar in both groups being 0.77 ± 0.19 in the D-DALK group and 0.75 ± 0.19 in the PD-DALK group (*P* = 0.75) (Table 1).

No differences in topographic postoperative corneal astigmatism were found between the two groups at 1 and 6 months after surgery.

### 3.1. IVCM Results

The deep lamellar stromal donor-host interface was clearly identified in all patients at IVCM examination after 1 month.

**Figure 2:**
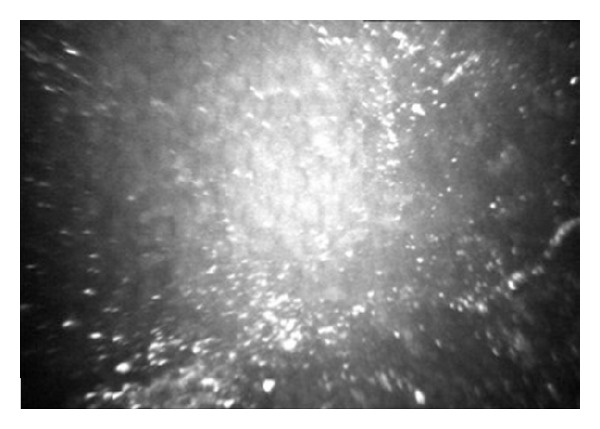
In vivo confocal microscopic image of a descemetic (D-DALK) interface. One month after surgery, a moderately hyperreflective and homogeneous layer, adjacent to the endothelium, was generally observed. Bright microdots are clearly visible in this case.

The interface morphological characteristics were found to be strictly dependent on the surgical technique. In the D-DALK group the interface was detectable as a uniform hyperreflective layer, adjacent to the endothelium presenting with variable transparency and the possible presence of bright microdots (Figure 2).

**Figure 3:**
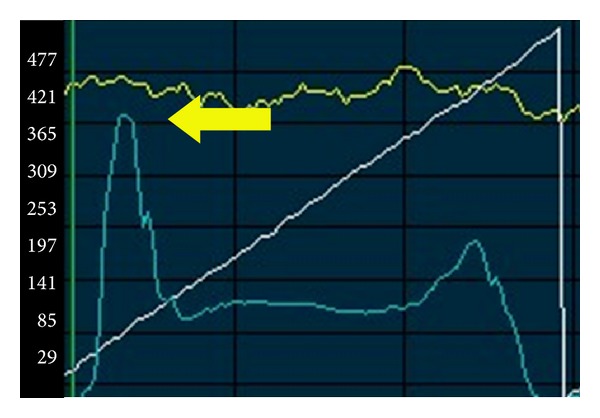
Z-scan curve representing a D-DALK 1 month after operation. The graph is characterized by a single highly reflective peak corresponding to the complex descemetic interface-endothelium (arrow).

Keratocytes nuclei were visible adjacently to the donor side of the interface. With respect to reflectivity values, in the Z-scan curve, a single highly reflective peak corresponding to the complex descemetic interface-endothelium was observed in all patients (Figure 3).

**Figure 4:**
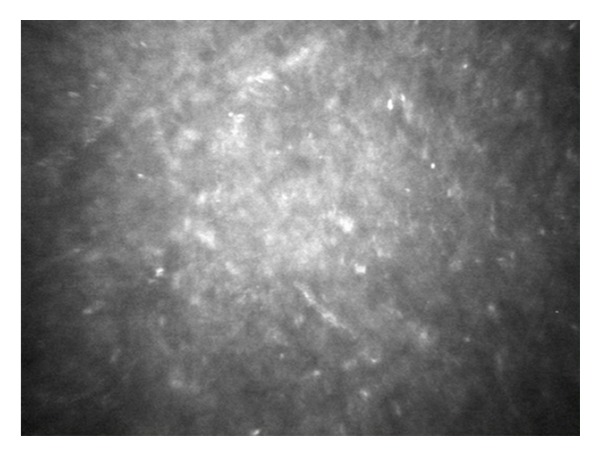
In vivo confocal microscopic image of a predescemetic (PD-DALK) interface 1 month after operation. A hyperreflective nonhomogeneous layer characterized by discontinuity in the deep stromal extracellular architecture was generally observed. The predescemetic interface appeared generally devoid of keratocyte cells and presented a variable degree of tissue oedema and hyperreflective extracellular matrix irregularities.

**Figure 5:**
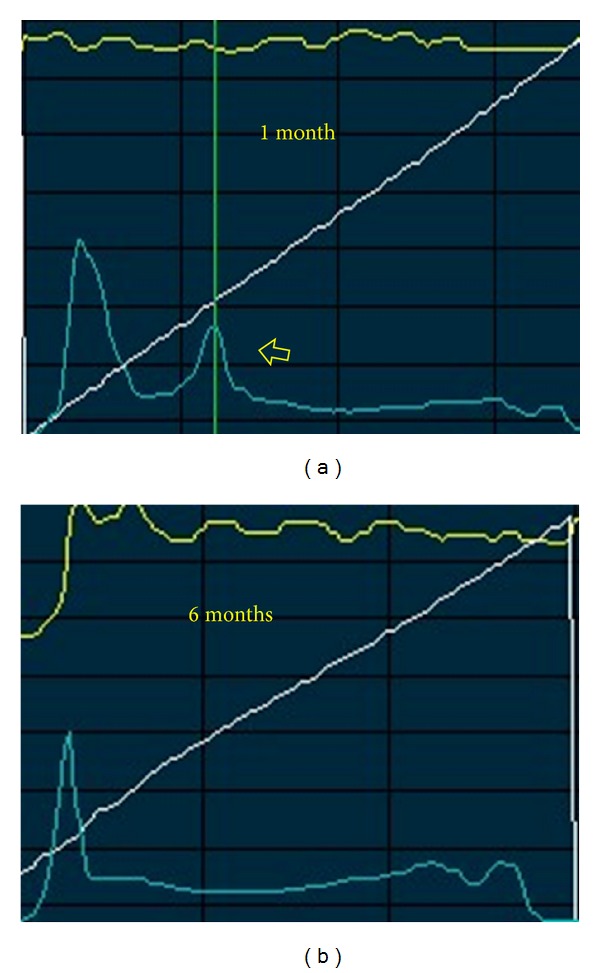
Z-scan curve representing a PD-DALK. (a) One month after surgery two different peaks of reflectivity are clearly identifiable and the deep stromal peak corresponding to the donor-host interface is located anteriorly to the endothelium-DM layers peak (arrow). (b) At 6 months after surgery the single interface peak is not visible anymore.

In the PD-DALK group the interface was represented as a discontinuity in the deep stromal extracellular architecture with evident reduction or absence of keratocytes in the stromal sublayers delimitating the proper interface and was associated with hyperreflectivity (Figure 4). Similarly to the D-DALK group, the presence of multiple bright microdots at the level of the interface was a frequent finding although not observed in all eyes. A variable quantity of donor stroma was identified between the interface and the DM. Accordingly, the Z-scan curve in PD-DALK group was characterized in the early stages by two peaks of reflectivity, one in the deep stroma corresponding to the donor-host interface located anteriorly to the endothelium-DM layers peak (Figure 5).

The interface morphological characteristics in the two groups of patients presented evident differences during the follow-up period.

The peak of reflectivity corresponding to the interface was 136.1 ± 36.8 LRU 1 month after D-DALK and was found to be unchanged at 6 months (132.6 ± 24.4 LRU, *P* = 0.52). The reduction of the number of bright microdots was the only detectable change during the follow-up period.

The peak of reflectivity 1 month after PD-DALK was 164.8 ± 36.9 LRU and it was significantly greater than the peak of reflectivity found 1 month after D-DALK (136.1 ± 36.8 LRU, *P* = 0.022) but it reduced significantly 6 months after surgery (136.2 ± 24.2, *P* = 0.0007) when it was found to be similar to the reflectivity of the D-DALK group (*P* = 0.7) (Table 1).

In the PD-DALK group the presence of keratocytes located adjacently to both sides of the interface with elongated shape and reflective cellular body “activated” was detected 6 months after surgery.

Residual bed thickness was not measurable in the D-DALK eyes since the interface was straight next to the DM (distance close to 0). In the PD-DALK eyes residual bed thickness had a great variability between patients. Average residual bed thickness was 122 ± 58 *μ*m (range 75–230 *μ*m) 1 month after surgery and 117 ± 54 *μ*m (range 65–220 *μ*m) 6 months after surgery.

## 4. Discussion

Visual recovery after deep anterior lamellar keratoplasty is influenced, among the other optical variables, by the transparency of the corneal tissue. Differently than penetrating keratoplasty in DALK the presence of a donor-host interface may influence the visual outcome of the patient due to abnormal light scattering of this layer or, in rare cases, interface haze [1]. While slit-lamp imaging of the DALK interface may only provide gross and subjective morphological details, in vivo confocal microscopy can provide fine imaging with high magnification at a cellular level. Moreover, it provides values of reflectivity of selected stromal layers, thus giving to clinicians a subtle analysis of the donor-host interface characteristics. At IVCM examination the optical transparency of the corneal layers is expressed by light reflectivity values and the lower is the reflectivity the higher is the corneal transparency.

Our results showed that one month after surgery the interface reflectivity, measured by confocal microscopy, was lower in patients where a successful baring of the DM was obtained (D-DALK group) compared to patients where some stromal tissue adherent to the host DM was left (PD-DALK group). This difference was no longer detectable at 6 months after operation, when the reflectivity was comparable in the two groups.

Microscopic interface features between the two groups showed distinctive morphological characteristics that can explain the different interface reflectivity observed in the two groups. At one month after PD-DALK technique the interface is characterized by a discontinuity in stromal extracellular architecture, with nonhomogeneous increased reflectivity due to tissue oedema and presence of activated keratocytes located in the stromal layers adjacent to the interface optical section. The presence of activated keratocytes and tissue oedema was reduced at 6 months and this may represent an index of tissue remodeling responsible for the observed decrease of reflectivity.

The irregularity of the host side stromal bed surface in the PD-DALK can justify the relative absence of keratocytes from the interface in the early postoperative period and consequently a longer recovery of transparency of the tissue.

Conversely the interface morphology in the D-DALK group, one month after operation, was characterized by a more homogeneous and lower reflectivity, with respect to the PD-DALK group. A mild degree of keratocyte activation was observed in the optical sections corresponding to the donor stromal layers adjacent to the descemetic interfaces. The proper interface appeared devoid of cells and with homogenous reflectivity. Activated keratocytes and bright microdots were reduced at six months follow-up, but the overall interface reflectivity did not change significantly. It is possible to hypothesize that D-DALK technique induces the formation of a more regular deep interface characterized by lower keratocyte response and tissue oedema and, therefore, by lower reflectivity in the early postoperative period, as compared to the PD-DALK surgery.

This phenomenon may explain, at least partially, the fact that the mean visual acuity in the early postoperative period (one month after surgery) was significantly greater in the descemetic keratoplasties with respect to the predescemetic cases. Conversely, visual acuity in the PD-DALK group significantly increased to reach the values of the D-DALK group at 6 months after surgery, indicating that visual acuity improved as the interface reflectivity recovered to normal values.

The result of our study is consistent with the previous literature concerning the evaluation of DALK by confocal microscopy.

Marchini et al. in 2006 reported how deep lamellar keratoplasty (DLKP) performed by intracorneal dissection provides visual and clinical results comparable with that of other DLKP techniques. They performed confocal microscopy to describe corneal features, interface morphologic features, and reflectivity, demonstrating a negative correlation between interface reflectivity and BCVA showing that the progressive recovery over months of the interface transparency is correlated with the increase in visual acuity after 6 months [11].

Abdelkader and Kaufman published in 2011 a perspective study on twenty eyes of 20 patients affected by corneal disease with healthy DM and endothelium treated with DALK. They report the visual outcome and the microscopical feature with a clinical evaluation of the interface haze. The patients were divided in two groups: 12 eyes had DM completely bared (descemetic group) and 8 eyes had a fine layer of nonpathological deep stroma left intraoperatively (predescemetic group). No statistically significant difference in mean visual outcomes was found between the groups at 6 months (*P* = 0.057). In the predescemetic eyes, the remaining fine layer of the stroma was clear and healthy. The interface haze, evaluated at the slit lamp, progressively declined over time and does not compromise the visual acuity. Since 1 week after surgery they described an increase of brightness of the interface due to activated keratocytes presence detected by confocal microscopy. The brightness and reflectivity of activated keratocytes at the interface were less intense in the descemetic group compared with the predescemetic group 7 days after the surgery. They report a progressive reduction of keratocyte brightness and reflectivity occurring in both the groups over time. At the end of the study keratocyte morphology and reflectivity had returned to normal. This healing process was reported to be faster in the D-DALK group (4–6 weeks) than in PD-DALK (10–12 weeks) group [8].

Several clinical studies compared the visual outcome of DALK, descemetic, and predescemetic with PK [2, 12–14].

Sugita and Kondo in 1997 reported no statistically significant difference in the visual acuity at 12 months after DALK among 80 eyes that had DM exposure and 40 eyes that had a fine layer of the deepest stroma left intraoperatively [15].

Sarnicola et al. in a retrospective study published in 2010 reported no difference in visual acuity between the PD-DALK and D-DALK groups at an average follow-up of 30.4 months, although the eyes in the D-DALK group seemed to have faster visual recovery [7].

Fontana et al. published in 2011 a retrospective, nonrandomized, comparative case series. Sixty patients (60 eyes) with advanced keratoconus underwent DALK using the big bubble technique. They found comparable visual function between DALK and PK when the recipient stroma is fully excised and the DM is exposed (D-DALK), whereas visual results are inferior to those of PK when layers of stroma are left adherent to the DM, with creation of a stroma-stroma interface (PD-DALK). They underline how “the presence of a graft-host interface potentially influencing visual function after DALK is of major concern for surgeons” and how “manual stromal dissection, carried out in the very deep recipient stroma, results in the formation of an interface of poorer optical quality, leading to inferior visual results when compared with PK.” The results of this retrospective study can be influenced by the different follow-up of each patient [6].

It is widely demonstrated in literature that DALK surgery can be a valid surgical option to obtain a good visual recovery in keratoconus patients with healthy endothelium [1–4].

The complete baring of the DM must be considered by the surgeon as the principal goal to achieve intraoperatively, and the results of our study suggest that the D-DALK leads to a faster visual recovery by creating less interface reflectivity and scarring. On the other hand, in our sample, the intraoperative incomplete baring of the DM does not compromise the long term visual results. In fact the healing process supported by keratocytes seems to take, after time, to an improve of transparency of the donor-host interface with recovery of a satisfactory VA.

## Figures and Tables

**Table 1:** One- and six-month results.

	Group	BCVA (Snellen)	BCVA (Snellen)	BCVA (Snellen)	Endothelial cell (Cell/mm^2^)	Endothelial cell (Cell/mm^2^)	Endothelial cell (Cell/mm^2^)	Interface reflectivity (LRU)	Interface reflectivity (LRU)	Astigmatism (D)	Astigmatism (D)
		Before operation	One month	Six months	Before operation	One month	Six months	One month	Six months	One month	Six months
Mean	Descemetic	0,32	0,55	0,77	2264,67	2227,00	2253,61	136,1	132,67	3,14	2,64
SD	Descemetic	0,17	0,18	0,19	429,16	380,09	328,14	36,8	24,44	1,88	1,29
Mean	Predescemetic	0,29	0,31	0,75	2135,75	2117,75	2134,17	164,8	136,17	3,38	2,63
SD	Predescemetic	0,12	0,7	0,19	387,53	363,12	372,76	36,9	24,24	1,55	0,86
